# Non-Destructive Inspection of High Temperature Piping Combining Ultrasound and Eddy Current Testing

**DOI:** 10.3390/s23063348

**Published:** 2023-03-22

**Authors:** David Santos, Miguel A. Machado, João Monteiro, José P. Sousa, Carla S. Proença, Fernando S. Crivellaro, Luís S. Rosado, Telmo G. Santos

**Affiliations:** 1 ISQ—Instituto de Soldadura e Qualidade, 2740-120 Porto Salvo, Portugal; 2 UNIDEMI, Department of Mechanical and Industrial Engineering, NOVA School of Science and Technology, Universidade NOVA de Lisboa, 2829-516 Caparica, Portugal; 3 Laboratório Associado de Sistemas Inteligentes, LASI, 4800-058 Guimarães, Portugal; 4 Bosch Termotecnologia S.A., 1800-220 Lisboa, Portugal; 5 Departament of Physics, NOVA School of Science and Technology, Universidade NOVA de Lisboa, 2829-516 Caparica, Portugal; 6 Instituto de Telecomunicações, 1049-001 Lisbon, Portugal; 7 Instituto Superior Técnico, Universidade de Lisboa,1049-001 Lisbon, Portugal

**Keywords:** ultrasonic testing, Eddy currents testing, pipelines, steel, weld bead, high temperature

## Abstract

This paper presents an automated Non-Destructive Testing (NDT) system for the in-service inspection of orbital welds on tubular components operating at temperatures as high as 200 °C. The combination of two different NDT methods and respective inspection systems is here proposed to cover the detection of all potential defective weld conditions. The proposed NDT system combines ultrasounds and Eddy current techniques with dedicated approaches for dealing with high temperature conditions. Phased array ultrasound was employed, searching for volumetric defects within the weld bead volume while Eddy currents were used to look for surface and sub-surface cracks. The results from the phased array ultrasound results showed the effectiveness of the cooling mechanisms and that temperature effects on sound attenuation can be easily compensated for up to 200 °C. The Eddy current results showed almost no influence when temperatures were raised up to 300 °C.

## 1. Introduction

Non-Destructive Evaluation (NDE) is continuously improving and changing. New techniques and challenges arising from new materials and manufacturing processes are continuously pushing Non-Destructive Testing (NDT) forward [1,2]. One good example concerns in-service high-temperature component inspection, whose industrial relevancy is easily sustained [3]. Components such as steam transportation pipelines must withstand high temperatures above 300 °C, requiring adapted in-service inspection and condition monitoring methods. There are several solutions for pipe inspection [4], but few application examples can be found for when high temperature conditions apply and where customized equipment is required [5,6,7,8].

The literature reports high-temperature NDT applications including permanent inspections of hot wire [5] and in-situ monitoring using Eddy currents [9] and ultrasound [10,11]. In these works, equipment was developed and/or adapted to answer the specific requirements of the end application, and could not be adapted to other uses.

High-Temperature Ultrasonic Testing (HTUT) has been continuously developed, driven by substantial industrial interest. Ultrasound Testing (UT) allows the inspection of optically opaque materials such as liquid-metal coolants, steam generator piping, and heat exchanger pipes [10]. However, the temperature at which ultrasonic transducers with piezoelectric elements may operate is limited by their Curie temperature [10,12]. The maximal operation temperature is also limited by the properties of the material used for the transducer’s assembly. Another aspect to consider is the temperature increase effect during increases in wave attenuation [11,13]. Research has focused on finding HTUT suitable couplants, yet high temperatures remain a demanding condition for probes and wedges, leading to their rapid degradation [14,15].

Other works have focused on temperature effects on UT results: A sparse-array including nine transducers for guided-waves has been applied to complex metallic structures; tests with temperature variations up to 8 °C and 2 °C spatial temperature gradients have been successfully demonstrated [16]. Two different temperature compensation methods have been evaluated—namely, optimal baseline selection (OBS) and baseline signal stretch (BSS). Both OBS and BSS have been demonstrated to be robust and practical solutions for temperature compensation [17].

Unlike sound properties, inspected material electromagnetic properties vary less widely with temperature. Therefore, Eddy Current Testing (ECT) [18] is a natural candidate, bringing benefits such as simple results interpretation and high testing speeds [19,20]. For ECT, the high-temperature challenge shifts to proper probe thermal isolation and the consequent lift-off. The temperature effect on ECT measurements has been reported in the literature; some researchers have looked at characterizing and/or compensating for small temperature variations to achieve enhanced accuracy [21]. One example is the inspection of fuel rods and plates employed in nuclear reactors [22], where the temperature effect has been modelled with empirical functions. In other examples, the inspected part undergoes high temperature variations—for instance, during heat treatment processes [23].

The ability to perform high-temperature inspections allows industrial components to be monitored during regular operations, reducing industrial shutdown periods and associated costs. The main objective of this work was to develop innovative inspection capabilities for critical components operating under high temperatures and pressures. Additionally, the combination of different testing methods is also proposed, granting complementarity and potential redundancy for the detection of defects at different locations and morphologies [24].

The paper and paper pulp industry were chosen as the demonstrative industrial end application. The proposed system allows the inspection of critical components while operating over temperatures high enough to prevent the application of traditional approaches and NDT equipment. The established temperature range covers the conditions of representative critical components, whose in-service inspection brings important benefits including down-time reductions and more efficient conditions control.

## 2. Experimental Setup and Samples

### 2.1. Standard Block

The developed standard block had a tubular geometry with two butt welds. The block base material was a class P235GH steel as specified in EN 10216-2 [25], suitable for high temperature and/or high pressure operating conditions. The standard block butt welds were carried out according to the specification WPS.141.01. These welds are characterized by a root step, applying the TIG method, proceeded by filling using coated electrodes. The manufactured standard block and the corresponding dimensions are presented in Table 1.

After performing ultrasound and X-ray NDT to ensure that no unintentional defects existed in the weld, three artificial defects were manufactured. These three defects—a weld toe notch, a weld root notch, and a weld bevel flat bottom hole—were chosen to represent traditionally found defects according to the paper pulp factory owner, and their characteristics were based on the standard blocks specified in ISO 13588 [26]. The artificially manufactured defects are specified in Table 2.

### 2.2. Automated Inspection System

To perform inspections on high-temperature components with contact-requiring techniques, it is crucial to use a semi/full automated system. The system was developed to ensure proper ultrasonic/Eddy current probe contact and positioning over the weld bead and to maximize the operator’s distance from the high-temperature component.

**Figure 1:**
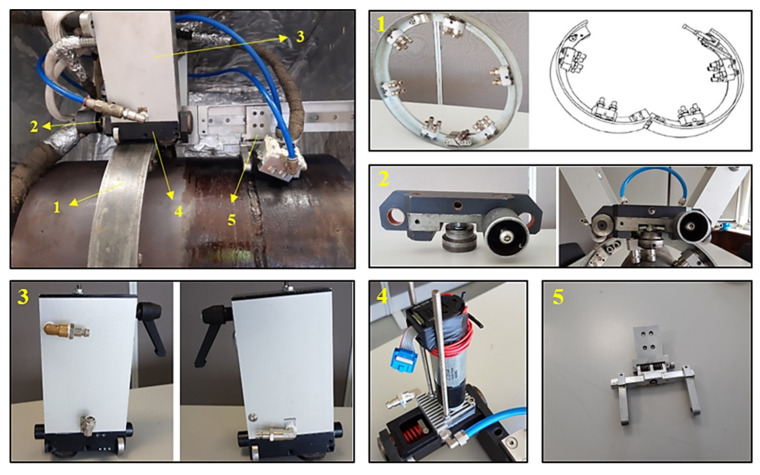
Automated inspection system elements.

The automated inspection system comprised a scanner attached to an orbital guide that was installed in the vicinity of the weld that was to be inspected. The guide included a hinge mechanism to allow for simple and fast installation around the pipe. Different probes could be mounted in the scanner using dedicated holders for ultrasonic and Eddy current probes. Figure 1 shows the scanning system mounted on the standard block.

The inspection system was composed of five main features:

- Orbital guide around the pipe, enabling the scanner’s movement—element 1;
- Quick tightening joint, to attach the scanner to the orbital guide—element 2;
- Air-cooled housing, to shelter the DC motors—element 3;
- Water-cooled base plate, where the DC motors were fixed—element 4;
- Probe holder—element 5.

The orbital guide around the pipe (Figure 1, element 1) consisted of two halves, with a swivel joint at one end and a fastening system with only one screw at the other end. The guide supports to the tube were adjustable, allowing the same guide to be used in different tube diameters within a range of 30 mm. A quick tightening joint—Figure 1, element 2—allowed for engagement of the scanner in the guide by manually tightening a screw.

The driving system gear engaged the orbital guide (with toothed sides), ensuring power transmission and avoiding landslides due to the scanner’s own weight. The body of the scanner consisted of two housings—Figure 1, element 3—each incorporating a DC motor coupled to a toothed drive wheel. This system was cooled by compressed air inside the housing. The DC motors base plate was water-cooled as in Figure 1, element 4. The two brushed DC motors were a A-max 32 model from Maxon^®^ as well as the positioning controller EPOS2 24/5.

**Figure 2:**
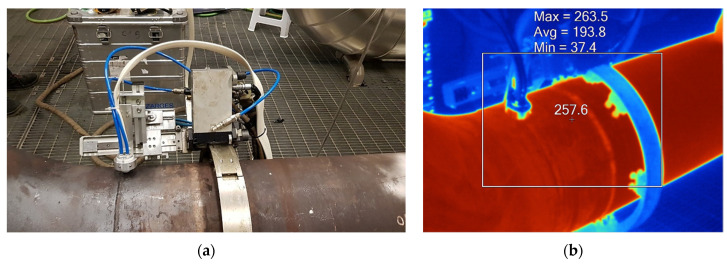
ECT system Validation: (**a**) automated inspection system coupled with ECT probes while performing a scan along the pipe perimeter; (**b**) thermographic image with temperature represented in degrees Celsius (°C).

The pipe temperature was monitored during the high-temperature inspections with a thermographic camera Fluke Ti400 with an emissivity index of 0.95, as depicted in Figure 2.

### 2.3. Heating and Temperature Control

**Figure 3:**
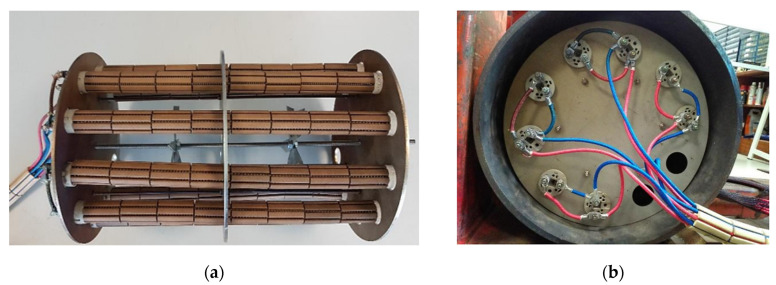
(**a**) Heating system assembly and (**b**) electrical wires for the resistances.

To perform the laboratorial experimental tests with the standard block at high temperatures, a heating system was designed and manufactured. Heat was generated through eight 1000 W electrical resistances driven by 4 rheostats (each rheostat controlled 2 electrical resistances), inserted inside the standard block as shown in Figure 3.

Heat regulation was performed by adjusting the rheostats and controlling the electrical resistances, dissipating the power. To conduct the tests, small increments of electrical power were made to achieve a total of 8 temperature steps from room temperature to 200 °C. A 2 h interval was maintained between increments so that the standard block temperature could stabilize before the experimental tests. Temperature control and recording was carried out with a thermometer (Tempil IRT-16, temperature range −60 °C to 625 °C).

## 3. Ultrasound Testing

### 3.1. Probe and Wedge Concept

**Figure 4:**
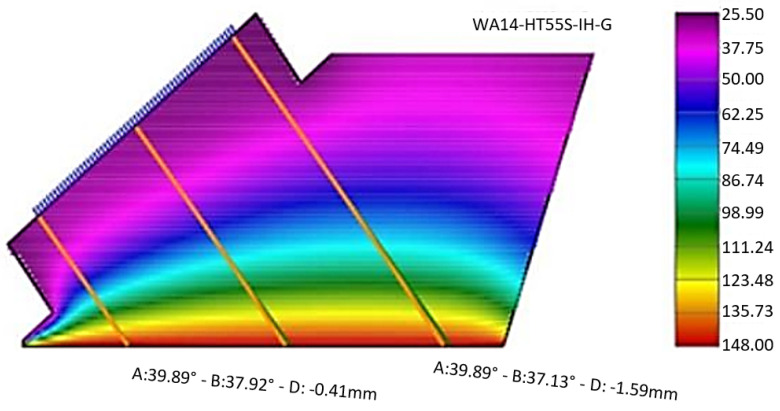
Beam variation with temperature.

It was considered that temperature increases have two direct consequences on ultrasound inspections: increased attenuation and increased propagation speed. It is possible to reduce the attenuation of sound waves by using lower-frequency probes; however, it must be noted that this also lowers the sensitivity. Concerning propagation speed increases, these can be easily measured and corrected in the used instrumentation. The real problem occurs when the test piece presents a temperature gradient along the propagation direction of the sound beam, as in most practical situations. This temperature gradient affects the sound wave velocity—thus causing the sound beam deviation. Figure 4 depicts a phased array shim with a temperature gradient; the orange line represents the beam direction in normal conditions while the green line is the deviation suffered due to the temperature gradient. This deviation is relatively small, although this effect is amplified as sound waves propagate through the piece.

Distinct phased array probes were studied with the simulation software EXTEND CIVA 2017, looking for the best options. A 20-element linear phased array probe IMASONIC^®^ 12051 operating at 3.25 MHz was selected, providing enhanced flaw responses (in terms of signal amplitude) and signal to noise ratios. The probe specifications are presented in Table 3.

**Figure 5:**
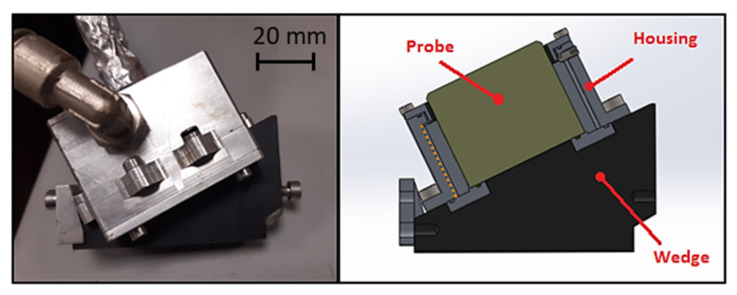
US Probe and wedge assembly.

The selected phased array probe had a maximum operating temperature of 60 °C; therefore, a water-cooled housing was required to ensure that the wedge top and the probe remained cool during operation. Figure 5 presents the adopted wedge and probe configuration.

Additionally, wedges made of heat-resistant materials were tried in order to cope with the high temperatures. A wedge produced in Duratron^®^ PBI (Polybenzimidazole) was produced to withstand temperatures of up to 200 °C. This polymer material, developed by Quadrant Plastics, has announced temperature services at up to 310 °C continuously and up to 500 °C for short periods of time. Another characteristic of this material is its low thermal expansion coefficient and constant material properties up to 400 °C [9].

The wedge material had a longitudinal ultrasound velocity of 2970 m/s and a 25.7° wedge angle, producing a 60° central angle in the standard block. An appropriate couplant was necessary to ensure good and constant ultrasound transmission between the probe and the piece. The couplant must have high wettability, corrosion resistance and proper viscosity and removal properties. Since traditional water and gel-based couplants cannot be used at high temperatures, a vegetable oil was used instead, after verifying that it is non-flammable at the involved temperatures nor produces irritant or harmful smokes for the operator.

### 3.2. Focal Laws

For each produced artificial defect, a dedicated focal law was defined to enhance detectability in each zone of the weld. These focal laws were defined bearing in mind ISO 17640 [26] and ISO 13588 [27]. A sectorial scan ensures normal wave incidence with a half skip to the bevel and accounts for minor deviations in the weld bevel angle.

A combination of sectorial scanning with electronic scanning was used for the flat bottom hole in the bevel (simulating a lack of fusion). In this case, longitudinal ultrasound waves with half-skips are used to generate the desired angles and to ensure complete coverage of the weld. The notch in the weld toe simulates a crack next to the weld or in the Heat Affected Zone (HAZ), so a half-skip is also needed to obtain the desired coverage. In this case, transversal ultrasound waves were used, with a combination of sectorial and electronic scanning. Simulating cracks in the root, the detection of this notch was made through longitudinal waves in direct mode (no skips), as well as with a combination of electronic and sectorial scanning. Three focal laws were defined—one for each defect type. Version 6.9.25 of the Multi2000 software and MULTIX++ hardware, both from the company M2M, were used for data acquisition and processing. Table 4 presents the parameters of each focal law, as well as the corresponding beam coverage.

### 3.3. Scan Plan

**Figure 6:**
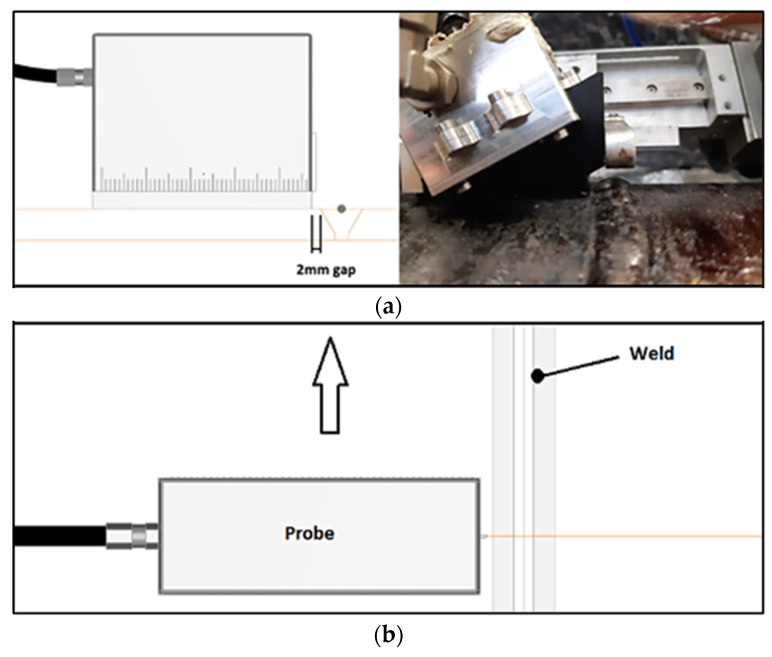
Scan plan. (**a**) Probe position; (**b**) Probe movement.

The previously defined focal laws were calculated for a probe relative to weld positioning as shown in Figure 6, with a 2 mm offset between the wedge front face and the weld toe. This 2 mm gap exists to account for small geometric irregularities that the weld cap may exhibit. The mechanical probe movement in relation to the weld is only longitudinal—an advantage of using phased array technology. Data acquisition for each defect was made independently, with a 60 mm length scan.

### 3.4. Experimental Results

When using ultrasounds for high-temperature inspections, one must account for both velocity variations and increased attenuation. A recalibration process is required to assess real velocity values. In steel, velocity changes by about 1% for each 55 °C variation in temperature [28]. In opposition, sound attenuation increases at higher temperatures. In typical fine grain carbon steel alloys, at room temperature and 5 MHz frequency, attenuation is approximately 2 dB per 100 mm one-way sound path. Due to the higher attenuation, it is often necessary to increase the instrument’s gain to reach proper sensitivity [28,29]. Moreover, distance/amplitude correction (DAC) curves or TGV (Time Varied Gain) programs that are established at room temperature require adjustments.

**Figure 7:**
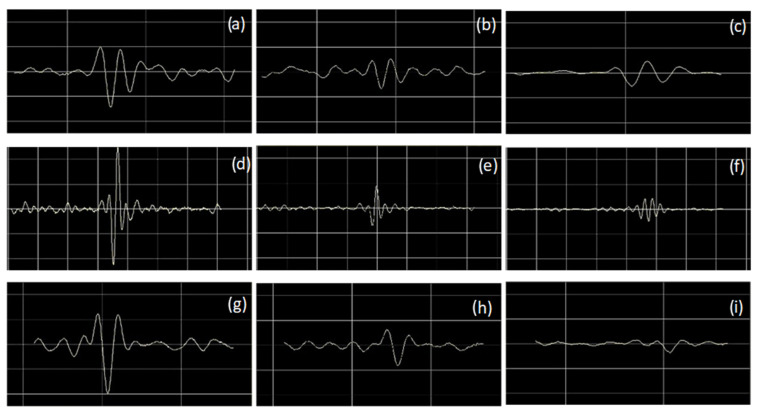
A-Scans obtained for: (**a**) weld root notch at 18 °C, (**b**) weld root notch at 122 °C, (**c**) weld root notch at 200 °C, (**d**) weld toe notch at 18 °C, (**e**) weld toe notch at 120 °C, (**f**) weld toe notch at 203 °C, (**g**) lack of fusion at 18 °C, (**h**) lack of fusion at 120 °C, and (**i**) lack of fusion at 201 °C.

Figure 7 shows the signal amplitude variation obtained for different temperatures tested for different defects. In the results obtained, it is possible to observe that the signal amplitude decreased with increases in temperature.

**Figure 8:**
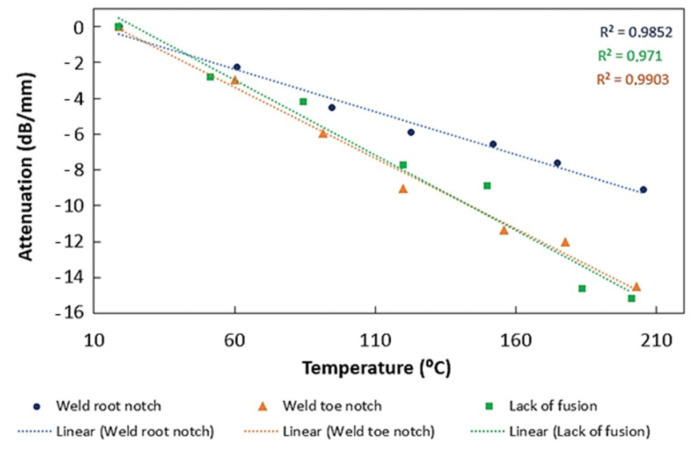
Variation in attenuation with temperature, for different defects.

**Figure 9:**
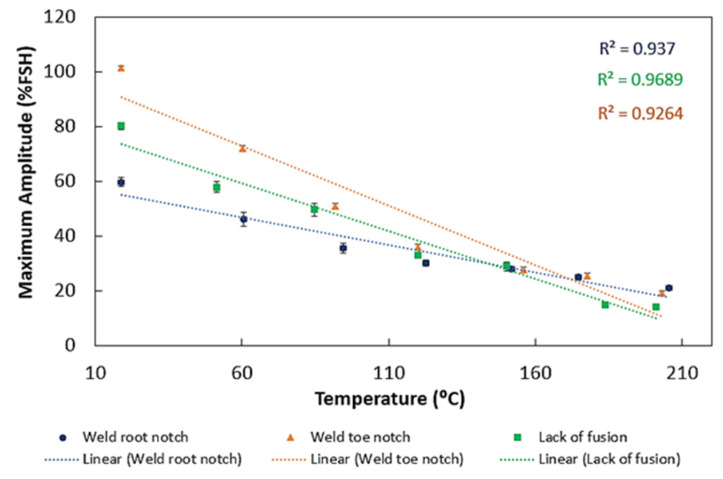
Variation of the maximum amplitude with the temperature for different defects.

Figure 8 shows the attenuation change with temperature for different defects. In all cases, it is possible to observe an attenuation decrease with higher temperatures. This decrease is most evident for the weld toe notch and lack of fusion defects. These results support those obtained for the maximum amplitude variation with temperature for different defects—Figure 9. As shown, as attenuation increased with higher temperatures, the signal amplitude also reduced; however, the decrease was lower for the weld root notch.

**Figure 10:**
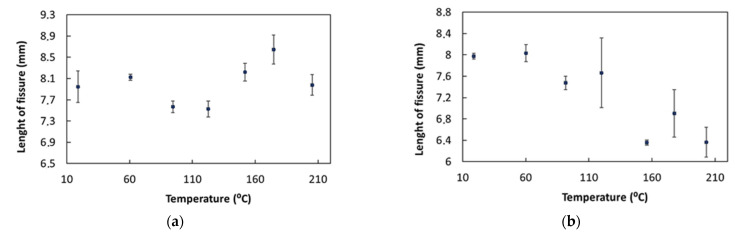

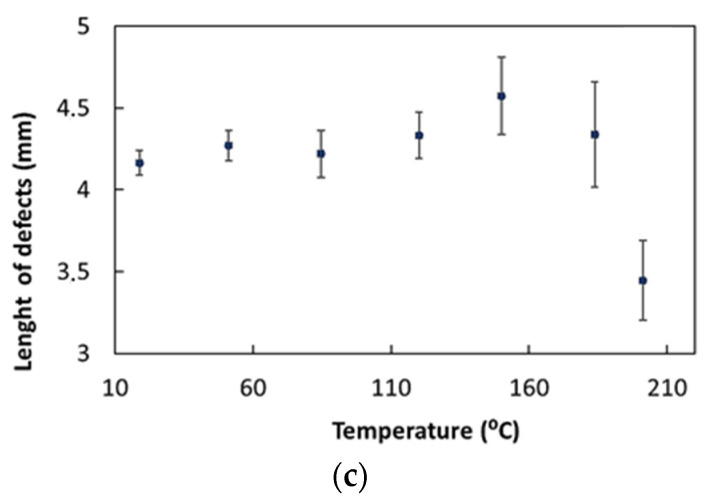
Variation in the length of defects with temperature, for (**a**) weld root notch, (**b**) weld toe notch and (**c**) lack of fusion.

From the obtained results analysis, it is possible to verify apparent variations in the defects’ dimensions along with temperature variations. Figure 10 shows the defects’ apparent length variation with increases in temperature. One possible explanation is, for instance, a compromised contact between the ultrasonic transducer and the piece due to the different thermal expansion coefficients of the two materials [30,31]. For the defect weld root notch, there was an irregular variation as the temperature increased. For the weld toe notch defect, it was possible to observe some irregularity, as the size of the defect decreased as the temperature increased. However, this apparent defect dimension variation was lower for lack-of-fusion defects up to temperatures of around 200 °C. At the highest evaluated temperatures, a decrease in the value of the defect dimension was observed. The major error source was related to the ultrasonic velocity being a function of temperature, with variations in the ultrasonic wave velocity at high temperatures [30]. Measurement errors can be minimized using a moving gate control with temperature variations, normalization of the signal amplitude, automatic determination of the ultrasonic flight time, and temperature compensation.

## 4. Eddy Current Testing

### 4.1. Customized EC Probes

**Figure 11:**
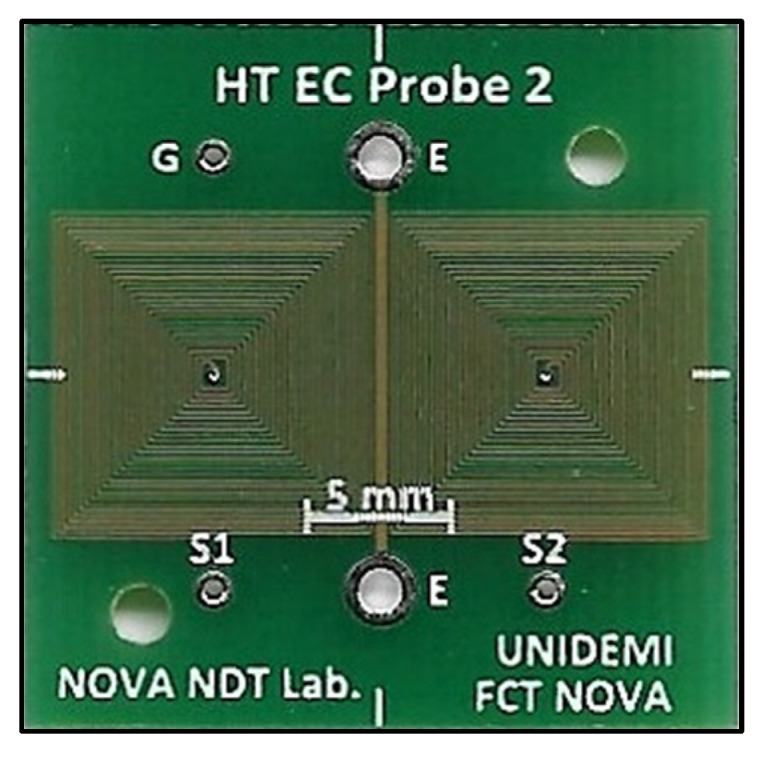
PCB probe developed for the inspection of the pipe base material. Linear excitation element cantered within the two differential coils.

Two customized ECT probe prototypes were developed—one targeting the defects’ detection on the pipe base material and the other targeting the defects’ detection on the weld bead. The first developed probe comprised two rectangular planar coils operating in a bridge differential mode. The planar disposition allowed a closer contact of all the coil’s windings with the material being inspected, increasing the probe’s sensibility. The coils were produced on Print Circuit Board PCB technology, which enables the production of planar geometries, can be automated, is economical, and brings enhanced reproducibility. Each coil was composed by 25 turns, with a track width and spacing in-between of 0.1 mm—Figure 11.

Since inspections have to be performed at high temperatures over long periods of time, the selected PCB base material was a high-performance FR-4 epoxy core and prepreg laminate with a glass transition temperature (Tg) of 180 °C. However, since piping must be inspected at even higher temperatures, water cooling was applied to reduce thermal effects.

**Figure 12:**
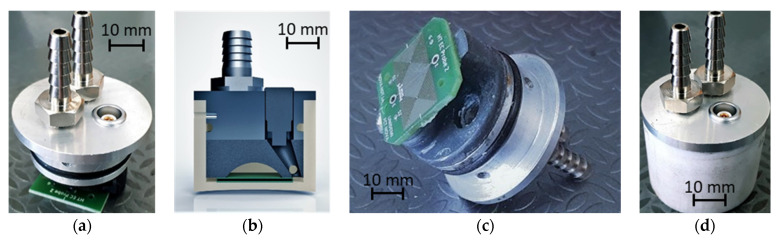
Construction process and chassis parts for the probe developed for the inspection of the pipe base material at high temperature. (**a**) PCB probe and lid assembled; (**b**) probe CAD cross section; (**c**) PCB probe and lid (view from bottom); (**d**) Assembled probe.

Targeting a robust, modular, and adaptable probe, a probe chassis with water cooling was developed. The chassis was composed of an insulating cup and a metallic cover. The PCB probe was fastened to an aluminum cover, where the probe connector was attached—as well as two water inlets for the refrigeration flow. For the cup, an insulating Teflon machined cup was developed. The construction process is detailed step-by-step in Figure 12, where: (a) shows the aluminum cover with the sealing O-ring and connectors for cooling and signaling; (b) and (c) the support for interconnecting the cover and the PCB; and d) the probe’s overall assembly-ready insert in the insulating cup.

**Figure 13:**
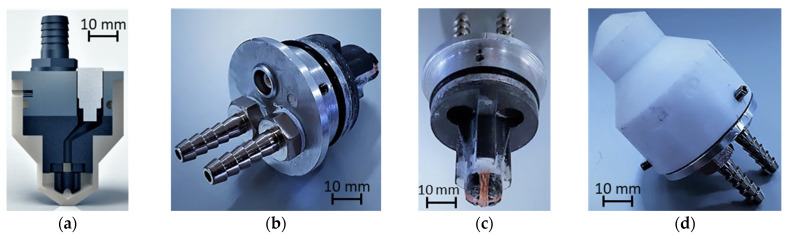
Construction process and chassis parts for the probe developed for the inspection of the pipe weld bead at high temperatures. (**a**) probe assembly cut-out in CAD; (**b**) the lid with the sealing O-ring and connectors for cooling and signaling; (**c**) the FDM-printed support used to hold the orthogonal coils; (**d**) the final external aspect of the probe.

The second probe targeted the detection of weld bead defects and was also composed of two coils in bridge-differential mode. The probe was designed so that the coils remained in the same position along the bead and maintained a similar influence over the weald bead on the two coils’ signals. This property allows for the reduction of the probe response to weld bead profile variations that otherwise mask the typically small response to lowest-dimension defects. As the defect orientation is naturally unknown, the probe must be also sensitive to defects with any alignment with respect to the weld bead. The adopted solution was the orthogonal crossing coils shown in Figure 13, which are often employed in the inspection of other welded parts.

A similar construction strategy as the one in the first probe was adopted. Figure 13 shows the developed chassis in each assembly step: (a) shows the overall probe assembly cut-out; (b) the lid with the sealing O-ring and connectors for cooling and signaling; (c) the FDM-printed support used to hold the orthogonal coils, and (d) the final external aspect. The external shape of the cup can also be seen in Figure 13, which was specially developed to produce the correct positioning of the probe before the welding.

### 4.2. Experimental Results

The customized Eddy current probes were experimentally validated using the automated inspection system presented in Section 2.2, Figure 2a.

#### 4.2.1. Base Material Inspection

**Figure 14:**
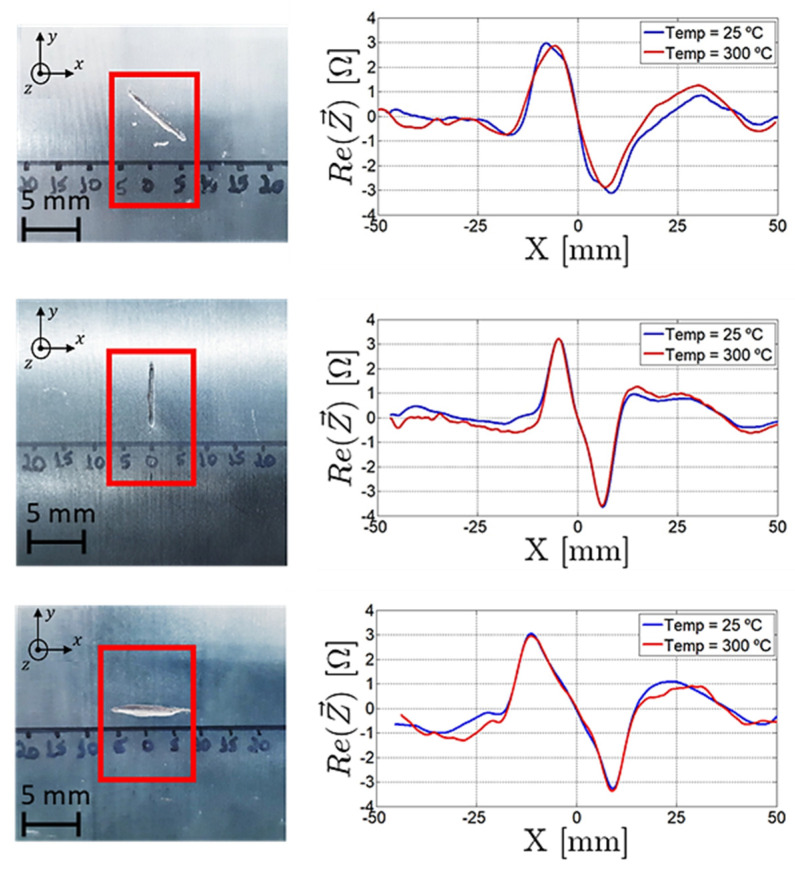
Output signal of the base material probe when scanning through the artificially made defect with different orientations.

For the experimental validation of the base material probe in Figure 12, three rectangular defects were artificially produced, being 0.5 mm wide, 0.4 mm deep and 10 mm high and with an angle of 0°, 45° and 90° in a 329 mm diameter 16Mo3 steel pipe. The gathered results shown in Figure 14 were obtained at 1 MHz frequency while the probe travelled in the X direction, centered with the defect. Through the superposition of the signals, the characterization of the defects did not suffer from temperature increases, which indicates that the probe cooling was effective.

**Figure 15:**
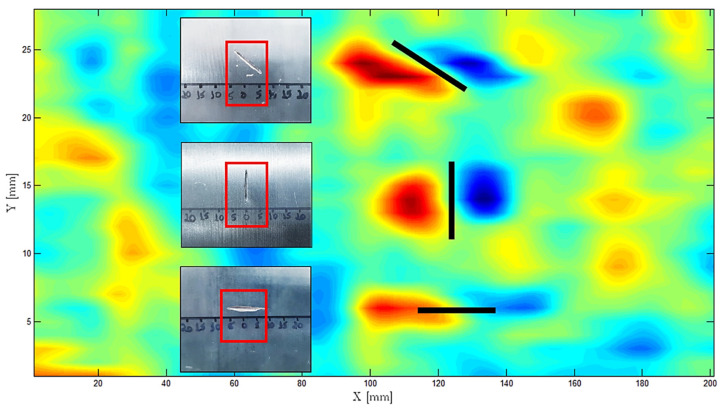
C-scan output signal operating the PCB probe for pipe base material at 1 MHz at 240 °C.

One-dimensional scans are useful for studying probe signal characteristics; however, C-scans are required to cover the whole area of the inspected pipe. Figure 15 shows the output signal in a C-scan performed over a 200 mm × 30 mm area containing the same defects. All three defects were clearly detected in the C-scan, which additionally provided some insight into the defects’ morphologies, orientations, and dimensions.

#### 4.2.2. Weld Bead Inspection

**Figure 16:**
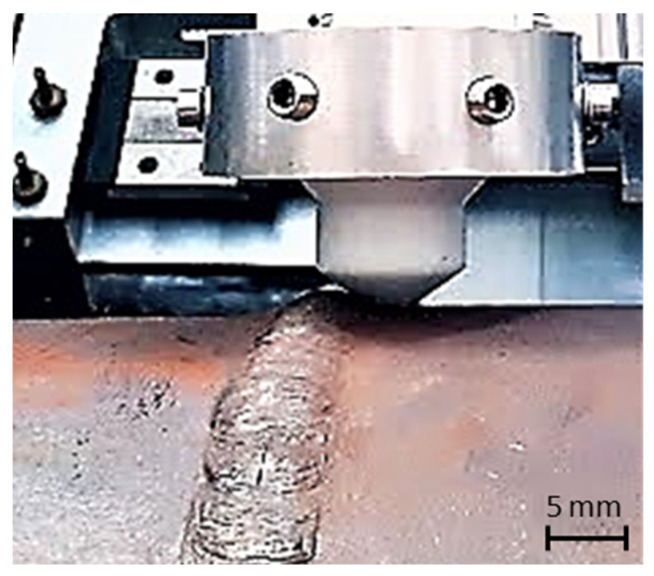
ECT probe developed for the weld bead inspection performing a scan on the bead’s right side.

Inspections were carried out on the two artificially manufactured defects on the outer surface of the tube. Scans along the perimeter of the tube were carried out on both the right and left sides of the weld bead, Figure 16. Measurements were recorded continuously while the automated inspection system was moving along the pipe perimeter. The excitation frequency used was 300 kHz in all tests.

**Figure 17:**
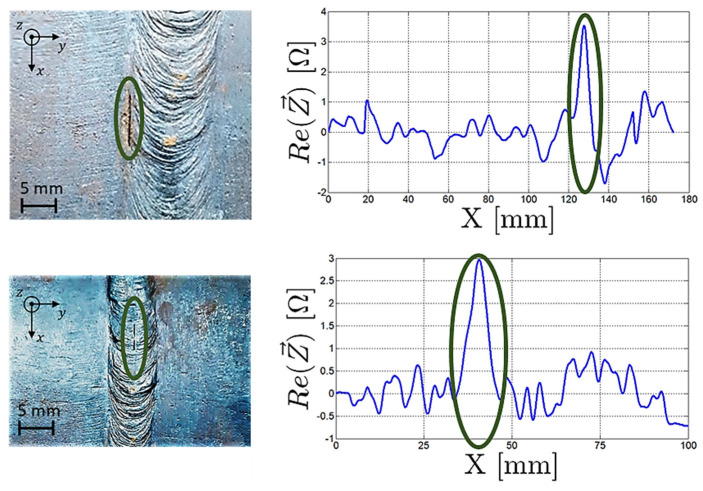
Output signal of the weld bead probe when scanning along the weld bead interface and centre.

Figure 17 shows the defects present in the weld beads—approximately 8 mm long, 1 mm wide, and 3 mm deep. One centered with the weld bead and one was at the weld bead’s border with the base material. Besides the background signal resulting from the weld bead surface profile variations, a signal peak caused by the defects’ presence stood out.

## 5. Conclusions

This work demonstrates the application of UT and EC for high-temperature in-service industrial components. Although the target application related to the paper industry, benefits could materialize across a wide range of industries including chemical and energy production. The ability to perform inspections while continuously operating reduces the frequency of planned outages. On the other hand, the possibility of monitoring known defects and degradation allows for the taking of informed decisions on whether or not interrupt operations and to better plan future interventions.

The UT probes’ designs for active cooling proved to be effective and favourable for good ultrasound propagation at the same time. The wedge also provided some thermal insulation, helping to keep the probe under operating conditions. The reported results showed the adopted solutions’ effectiveness up to 200 °C and that temperature effects can be compensated for by a moving gate control with temperature variation, normalization of the signal amplitude, automatic determination of the ultrasonic flight time, and temperature compensation.

Despite the high lift-off required for the high-temperature EC testing, both differential probes obtained very good results. The PCB probe showed improved results with a higher signal-to-noise ratio, and high temperatures did not have a significant impact due the effective cooling of the ECT probes. The produced prototypes allowed experimental validation, with results demonstrating almost no influence when temperatures were raised up to 300 °C.

## Figures and Tables

**Table 1:** Standard block specifications.

General Dimensions		Standard Block
Length	500 mm	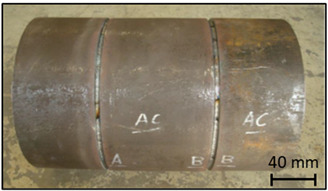
Thickness	7.1 mm	
Outer diameter	323.9 mm	
Weld dimensions		Weld cut view
Bevel type	60° V bevel	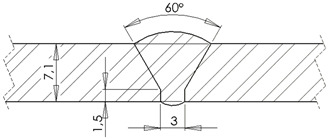
Root height	1.5 mm	
Root spacing	3 mm	

**Table 2:** Artificially manufactured defects characterization.

Defect	Weld Toe Notch	Weld Bevel Flat Bottom Hole	Weld Root Notch
Manufacturing method	EDM (electrical discharge machining)	Drilling	EDM (electrical discharge machining)
Length	8	-	8
Width	0.3	-	0.3
Height	1	-	1
Diameter	-	2.5	-
Position	Top surface, in the weld toe	Weld bevel (at 30°), centred at half thickness	Bottom surface, in the weld root
Representation	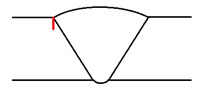	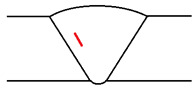	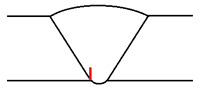

**Table 3:** Selected Phased array probe specifications.

N° of Crystals	Element Width	Gap between	Element Size	Central Frequency	Bandwidth
20	16 mm	0.2 mm	1 mm	3.25 MHz	60%

**Table 4:** Characteristics and beam coverage representation of each focal law.

**Weld bevel flat bottom hole**		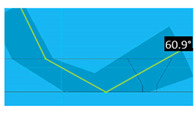
Wave type	Longitudinal	
Active elements	15 active elements 6 sequences	
Angle range	58.2° to 70.8°	
**Weld toe notch**		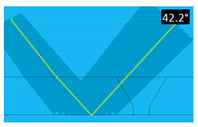
Wave type	Transversal	
Active elements	10 active elements 4 sequences	
Angle range	38.5° to 45.9°	
**Weld root notch**		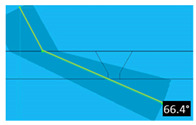
Wave type	Longitudinal	
Active elements	12 active elements 9 sequences	
Angle range	64.2° to 75.7°	

## References

[1] Ferreira P.M., Machado M.A., Carvalho M.S., Vidal C. (2022). Embedded Sensors for Structural Health Monitoring: Methodologies and Applications Review. Sensors.

[2] Santos T.G., Oliveira J.P., Machado M.A., Inácio P.L., Duarte V.R., Rodrigues T.A., Santos R.A., Simão C., Carvalho M., Martins A. (2020). Reliability and NDT Methods. Adv. Struct. Mater..

[3] Long Y., Luo J., Yue M., Wu G., Zhao M., Ji N., Song W., Jin Q., Kuang X., Fan Y. (2022). Investigation on leakage cause of 13Cr pipe flange used for a Christmas tree in a high-pressure and high-temperature gas well. Eng. Fail. Anal..

[4] Machado M.A., Rosado L., Pedrosa N., Miranda R., Piedade M., Santos T.G. (2017). Customized Eddy Current Probes for Pipe Inspection. Stud. Appl. Electromagn. Mech..

[5] Perez C. (2008). In-Line Quality Control of Hot Wire Steel—Towards Innovative Contactless Solutions and Data Fusion (Incosteel).

[6] Rahman M.M., Marklein R. Advanced Techniques for Modelling and Detection of Cracks in Hot Wire Steel. Proceedings of the 9th European Conference on NDT (ECNDT).

[7] Hartmann K., Ricken W., Becker W.-J., Pérez C., Gonzalo L. Improved Eddy Current Sensor for Hot Wire Inspection. Proceedings of the 9th European Conference on NDT (ECNDT).

[8] Ricken W., Hartmann K., Becker W.-J., Perez C., Gonzalo L. (2008). Optimierung von Wirbelstromspulen. Verbesserte Fehlerdetektion bei der Heißdrahtprüfung Optimised Eddy Current Sensor. Improved Defect Detection on Hot Wire Steel. TM Tech. Mess..

[9] Klümper-Westkamp H., Zoch H.-W., Reimche W., Bach F. (2011). High Temperature Resistant Eddy Current Sensor for “in situ” Monitoring the Material Microstructure Development of Steel Alloys during Heat Treatment—Bainite Sensor. Procedia Eng..

[10] Tittmann B.R., Batista C.F.G., Trivedi Y.P., Lissenden C.J., Reinhardt B.T. (2019). State-of-the-Art and Practical Guide to Ultrasonic Transducers for Harsh Environments Including Temperatures above 2120 °F (1000 °C) and Neutron Flux above 1013 n/cm^2^. Sensors.

[11] Kazys R., Vaskeliene V. (2021). High Temperature Ultrasonic Transducers: A Review. Sensors.

[12] Ferreira P.M., Machado M.A., Carvalho M.S., Vidal C. (2023). Granting Sensorial Properties to Metal Parts through Friction Stir Processing. Measurement.

[13] Slongo J.S., Gund J., Passarin T.A.R., Pipa D.R., Ramos J.E., Arruda L.V., Junior F.N. (2022). Effects of Thermal Gradients in High-Temperature Ultrasonic Non-Destructive Tests. Sensors.

[14] Netshidavhini N., Mabuza R.B. Effects of Various Couplants on Carbon Steel and Aluminium Materials Using Ultrasonic Testing. Proceedings of the 18th World Conference on Nondestructive Testing.

[15] Li C., Nordlund E. (1993). Effects of couplants on acoustic transmission. Rock Mech. Rock Eng..

[16] Clarke T., Cawley P., Wilcox P.D., Croxford A.J. (2009). Evaluation of the damage detection capability of a sparse-array guided-wave SHM system applied to a complex structure under varying thermal conditions. IEEE Trans. Ultrason. Ferroelectr. Freq. Control..

[17] Croxford A.J., Moll J., Wilcox P., Michaels J.E. (2010). Efficient temperature compensation strategies for guided wave structural health monitoring. Ultrasonics.

[18] Machado M.A., Rosado L.S., Santos T.G. (2022). Shaping Eddy Currents for Non-Destructive Testing Using Additive Manufactured Magnetic Substrates. J. Nondestruct. Eval..

[19] Machado M.A., Antin K.-N., Rosado L.S., Vilaça P., Santos T.G. (2021). High-speed inspection of delamination defects in unidirectional CFRP by non-contact Eddy current testing. Compos. Part B Eng..

[20] Machado M.A., Rosado L.F.S.G., Mendes N.A.M., Miranda R.M.M., dos Santos T.J.G. (2021). New directions for inline inspection of automobile laser welds using non-destructive testing. Int. J. Adv. Manuf. Technol..

[21] He P., Ma Y., Chen H. (2018). Temperature Drift Compensation of Eddy Current Sensor under High Temperature Environment. E3S Web Conf..

[22] Beck F.R., Lind R.P., Smith J.A. (2018). Temperature Sensitivity Study of Eddy Current and Digital Gauge Probes for Oxide Measurement. Res. Nondestruct. Eval..

[23] Vetterlein J., Klümper-Westkamp H., Hirsch T., Mayr P. September. Eddy current testing at high temperatures for controlling heat treatment processes. Proceedings of the International Symposium of Non Destructive Testing in Civil Engineering.

[24] Machado M.A., Rosado L.S., Mendes N.M., Miranda R.M., Santos T.G. (2021). Multisensor Inspection of Laser-Brazed Joints in the Automotive Industry. Sensors.

[25] (2014). *SRPS EN 10216–2: 2014*; Seamless Steel Tubes for Pressure Purposes. Non-Alloy and Alloy Steel Tubes with Specified Elevated Tem-Perature Properties. https://iss.rs/en/project/show/iss:proj:47290.

[26] (2019). Non-Destructive Testing of Welds, Ultrasonic Testing, Use of Automated Phased Array Technology.

[27] (2010). Non-Destructive Testing of Welds–Ultrasonic Testing—Techniques, Testing Levels, and Assessment.

[28] Burger B., Fuchs M. (2005). Introduction to Phased Array Ultrasonic Technology Applications.

[29] Dubé N. (2017). Advanced in Phased Array Ultrasonic Technology Applications: Olympus Guideline.

[30] Cheong Y.-M., Kim K.-M., Kim D.-J. (2017). High-temperature ultrasonic thickness monitoring for pipe thinning in a flow-accelerated corrosion proof test facility. Nucl. Eng. Technol..

[31] Oh S.-B., Cheong Y.-M., Kim D.-J., Kim K.-M. (2019). On-Line Monitoring of Pipe Wall Thinning by a High Temperature Ultrasonic Waveguide System at the Flow Accelerated Corrosion Proof Facility. Sensors.

